# The Proportion of Self-Reported Medication Administration Errors and Associated Factors: A Cross-Sectional Study

**DOI:** 10.3390/healthcare14131850

**Published:** 2026-06-25

**Authors:** Maram Banakhar, Nouran Katooa, Nada Alyoubi, Shatha Aloqmani, Rahil Alyoubi, Khulud Alsharif, Reem Al-Dossary, Noura Almadani

**Affiliations:** 1 Public Health Department, Faculty of Nursing, King Abdulaziz University, Jeddah 22254, Saudi Arabia; ahbbanakher3@kau.edu.sa; 2 Maternity & Child Health Department, Faculty of Nursing, King Abdulaziz University, Jeddah 22254, Saudi Arabia; 3 Faculty of Nursing, King Abdulaziz University, Jeddah 22254, Saudi Arabia; alyoubinada0@gmail.com (N.A.); shatha.m1421@gmail.com (S.A.); rahil776@gmail.com (R.A.); khuludalsharif505@gmail.com (K.A.); 4 Nursing Education Department, College of Nursing, Imam Abdulrahman Bin Faisal University, Dammam 34212, Saudi Arabia; rnaldosari@iau.edu.sa; 5 Community & Psychiatric Mental Health Nursing Department, College of Nursing, Princess Nourah Bint Abdulrahman University, Riyadh 11564, Saudi Arabia; naalmadani@pnu.edu.sa

**Keywords:** medication administration errors, nurses, patient safety, reporting practice, medication

## Abstract

**(1) Background**: Medication administration errors (MAEs) are potentially harmful incidents that may be avoidable. This study aimed to assess the proportion of self-reported MAE reporting among nurses in Saudi Arabia and to identify the associated factors. **(2) Methods**: A descriptive cross-sectional study was conducted among 259 nurses at a teaching hospital from January to March 2023. Data were collected via an electronic questionnaire and analyzed using descriptive statistics. **(3) Results**: The results of this study revealed that medication administration errors were reported at a higher level. The study demonstrated that nurses notify their department when a medication administration error occurs. The primary contributing factors identified for not reporting MAEs were high workload (84.1%) and fear of legal action (42.5%). Double-checking medications was the most recommended preventive measure (99.7%). **(4) Conclusions**: Recommendations to increase MAE reporting include workload management and fostering a non-punitive reporting culture to enhance patient safety.

## 1. Introduction

The overall proportion of medication administration errors (MAEs) was 22% according to the WHO systematic review [1,2]. Under-reporting of MAEs is one of the biggest problems in today’s healthcare [2]. Research shows that 5% of these errors result in death, and up to 50% can be prevented [3]. Recent developments in the field of health have led to a renewed interest in preventing MAEs [3], and researchers have shown a growing interest in knowing the causative agents [3,4]. MAEs occur when a patient receives a different medication from what the physician intended, errors related to dosage, route of administration, timing, or rate of administration, leading to potential harm [5].

A Jordanian study that aimed to review nurses’ reporting of such errors, determine the reasons for MAE underreporting, and evaluate the causes of MAEs from their perspective found that nurses had a favorable opinion of MAE reporting [6]. This study assists healthcare professionals in understanding the potential causes of error occurrence and the potential causes of MAE underreporting, because errors will always occur. In addition, a cross-sectional study conducted among 231 nurses in three hospitals in Kerman, Iran, revealed several significant findings regarding MAEs. Nurses identified high workload (79.2%), illegible handwriting by physicians (68.4%), nurse fatigue due to extended working hours (62.3%), and insufficient knowledge or training (47.6%) as the primary causes of medication administration errors. The study also highlighted notable barriers to reporting medication administration errors, including fear of punishment (56.3%), concerns about negative reactions from supervisors (50.6%), lack of familiarity with reporting procedures (48.5%), and fear of job loss (42.9%). These results underline the importance of addressing workload management, enhancing nurse training, and fostering a supportive and non-punitive environment to improve error reporting practices and patient safety [7]. Furthermore, an Ethiopian study found that the rate of reporting MAEs among 397 nurses was 57.4% [3].

Another study conducted in northwest Ethiopia employed a cross-sectional analysis of 282 nurses [8]. In this study, female nurses reported medication mistakes more frequently than male nurses, and this difference was statistically significant; in fact, male nurses were nearly three times less likely than female nurses to report MAEs [5].

Marital status was a significant predictor of reporting MAEs. Married nurses had a 54.6% lower likelihood of reporting MAEs than single nurses (AOR = 0.454; 95% CI: 0.251–0.821). Having made an error while administering medication was another crucial factor influencing the likelihood of reporting such errors. Nurses who had never reported a medication administration error were 55.5% more likely to do so than those who had [3]. A medication administration error may be reported more frequently if attitudes are changed to one of non-blame and anonymous reporting platforms are implemented [9].

A safety culture where nurses are encouraged and empowered to report errors or near-misses without fear of punishment—termed “Psychological Safety”—and receive feedback should be encouraged. Additionally, nurses should be informed about existing no-blame systems and anonymous reporting platforms. This will improve patient care by facilitating an understanding of the causes and variables contributing to medication administration errors. In another study, approximately 122 (54.5%) of the respondents cited unclear or illegible physician orders as one of the causes of MAEs, followed by frequent changes in physician orders (133 respondents (59.4%)), incorrect medication labeling by pharmacists (127 respondents (56.7%)), patients taking the same or similar medications (133 respondents (59.4%)), and inadequate training of unit staff on new medications (130 respondents (58.0%)) [5].

Among the factors that affect the non-reporting of MAEs are lack of time to complete the report, lack of knowledge of how the error occurred, low self-confidence, and lack of training [6,10]. Nurses refraining from reporting MAEs due to fear indicates concerns about the repercussions and places responsibility on the bosses and coworkers. This suggests that other nurses will not be aware of the potential harm caused to the patient and the lack of experience of the nurse. Another reason some nurses may choose to avoid reporting MAEs, which may increase the underreporting of administration errors, is the potential legal action that may result in them being fired from the hospital or having their wages garnished [5,7,8,9]. Research, education, policy, practice, and institutional and individual factors involved in medication administration errors were identified as the major barriers to safety [11]. Studies by reputable sources indicated that fear of reporting MAEs was prevalent among nurse practitioners, administrators, educators, and other healthcare professionals.

Lack of standardized reporting systems and fear of blame and punishment were also identified as significant barriers to MAE reporting in healthcare settings in various contexts [1,3,6,8,10,11,12,13,14,15,16,17,18]: fear of punishment (researchers, regulatory leaders, consumer/and disciplinary action) [1,11,15,16,17,18,19,20,21]; lack of a “just culture of safety”/lack of a no-blame culture [20,21,22,23,24]; fear of the press or media (licensing board/interdisciplinary collaboration and communication, nursing board) [21]; fear of losing one’s job [1,11,15,16,17,18,19,20,21]; nurses’ work environments that do not support safety, reactions from leadership, peers, and patients and their families; lack of front-line nurses’ voices in decision making [1,11,16,17,18,19,20,21,22,23,24,25,26,27,28]; fear of being considered a troublemaker [20]. There is a gap in the studies conducted in Saudi Arabia [9,14]. For example, most studies in this discipline were conducted more than ten years ago, had limited sample sizes, and the questionnaires used did not thoroughly explore the factors associated with willingness to report MAEs, given that all the survey questions were closed-ended. This study aimed to assess the proportion of MAE reporting among nurses and to identify the associated factors in reporting these errors in Saudi Arabia. The results of this study provide valuable insights into the current proportion of administration errors and their influencing factors among nurses in Saudi Arabia, ultimately contributing to the improvement in patient safety and quality of care in healthcare settings. Addressing this gap in research will help the relevant parties understand the challenges faced by nurses and facilitate the implementation of effective strategies to enhance medication administration practices and nurses’ willingness to report errors, enabling the creation of a safer healthcare environment for both patients and healthcare providers.

## 2. Materials and Methods

### 2.1. Study Design and Setting

A descriptive cross-sectional study was conducted at a teaching hospital in Saudi Arabia. This tertiary healthcare hospital provides comprehensive medical services to the public while maintaining quality standards across a wide range of medical specialties. The hospital includes more than 170 general and specialized outpatient clinics. The data was collected from January to March 2023.

### 2.2. Participants

The inclusion criteria included nurses who had been working at the teaching hospital for at least six months and were willing to participate in the study. Additionally, nurses who had made an error while administering medication within the past year were included in the sample. Physicians were excluded from the study. A convenience sampling technique was used to select the study participants based on the inclusion criteria. Data were collected from the nurses—who were sampled from all departments—using an electronic questionnaire.

### 2.3. Instrumentation/Data Collection Method

A survey consisting of 32 questions was distributed to all nurses in the department; responses were submitted simultaneously [6].

The questionnaire contained the following parts: (Part 1) Demographic characteristics of participating nurses (general information, including age, gender, education level, personal monthly income, marital status, work experience, number of patients admitted or discharged per day, number of shifts per month, and receipt of training on medication administration techniques and administration errors). (Part 2) Medication administration error reporting practice. This part included five statements measured using a 5-point Likert scale: “always, often, sometimes, rarely, or never.” (Part 3) Factors contributing to medication administration errors. This included 10 statements. (Part 4) Factors associated with the underreporting of medication administration errors. This included personal fear associated with reporting medication administration errors, nursing administration concerns, and the reporting process. (Part 5) Measures for preventing medication administration errors. This includes five statements in the questionnaire. Parts 3–5 were assessed using a 5-point Likert scale: “strongly agree, agree, neutral, disagree, or strongly disagree”. The internal consistency was assessed using Cronbach’s α (0.708 to 0.937).

Data were collected using a structured electronic questionnaire administered. The researchers approached nurses in each unit through the head nurse and invite them to participate in the study. Prior to participation, the purpose of the study and instructions for completing the questionnaire were clearly explained to all participants. In addition, explanatory information and guidance related to the questionnaire were provided to enhance clarity and ensure participants’ understanding of the survey items. The questionnaire was displayed directly on the iPad, enabling participants to complete the survey electronically.

### 2.4. Bias

This study relied on self-reported data, which may be subject to social desirability and recall bias. Anonymity was guaranteed to reduce response bias.

### 2.5. Sample Size

The sample size for the current study was determined using the Raosoft sample size calculator 2004 web version with a 95% confidence level and a 5% margin of error. A total of 259 nurses participated in the study, yielding a response rate of 100%.

### 2.6. Data Analysis

All statistical data were analyzed by using SPSS version 26 (IBM Corp., Armonk, NY, USA). Descriptive statistics summarized the study variables. Categorical data are presented as frequencies and percentages, while continuous variables are reported as mean ± standard deviation and median (interquartile range). The total scores were calculated for each of the six domains and categorized medication error reporting practice levels based on percentage scores. The normality of continuous variables was assessed using the Shapiro–Wilk test. Because the medication error reporting practice scores were not normally distributed, non-parametric tests were applied. The Mann–Whitney U test was used to examine the association between medication error reporting practice and socio-demographic characteristics. Additionally, Spearman’s rank correlation coefficient was employed to determine the relationship between medication error reporting practice and the five other domains: factors contributing to medication administration errors, factors associated with under-reporting, nursing administration concerns, the reporting process, and preventive measures. A *p*-value of <0.05 at a 95% confidence interval was considered statistically significant.

### 2.7. Ethical Considerations

Ethical approval was obtained from the teaching hospital and granted by the institutional review board committee (Approval No. HA-02-J-008) on 17 January 2023. All participation was completely voluntary, and questionnaires were distributed anonymously. Participants were assured that their information would be kept confidential.

## 3. Results

### 3.1. Demographic Characteristics

Table 1 describes the socio-demographic characteristics of the 259 nurses who participated in this study. 31.7% were between 31 and 35 years, with nearly all (87.6%) being female. A majority held a bachelor’s degree (57.5%). Most nurses reported a monthly income of 10,000 SAR or less (84.2%). Approximately two-thirds of the nurses were married (66.4%), and work experience was fairly evenly distributed, though slightly concentrated in the 6–10-year range (28.6%). Staffing ratios varied across departments. The most frequent patient-to-nurse ratios were 1:2 (25.1%) and 1:6 (22.8%), showing that workload intensity varied. Most nurses (71.8%) worked over 15 shifts per month, suggesting their schedules were demanding. Nearly all nurses (88.8%) reported receiving training in administrative techniques and error management, showing the institution values training.

### 3.2. Assessment of Medication Administration Error Reporting Practice

Table 2 indicates that nurses demonstrate generally high levels of medication administration error reporting practices. The mean scores for the first three items exceeded 4.6, suggesting that most nurses “often” or “always” report errors, irrespective of patient harm or the potential for system improvement. Notably, 89.2% of nurses reported that they “always” report errors to their department, with a mean score of 4.71 ± 0.91.

In contrast, reporting behaviors were more variable in situations involving repeated errors or when nurses were not directly involved. With regard to the statement “*I report medication administration errors only when similar errors have occurred previously in the department*”, a polarized distribution was revealed, with 39.8% of respondents indicating “never” and 41.3% indicating “always,” resulting in a lower mean score of 3.05 ± 1.83.

**Figure 1:**
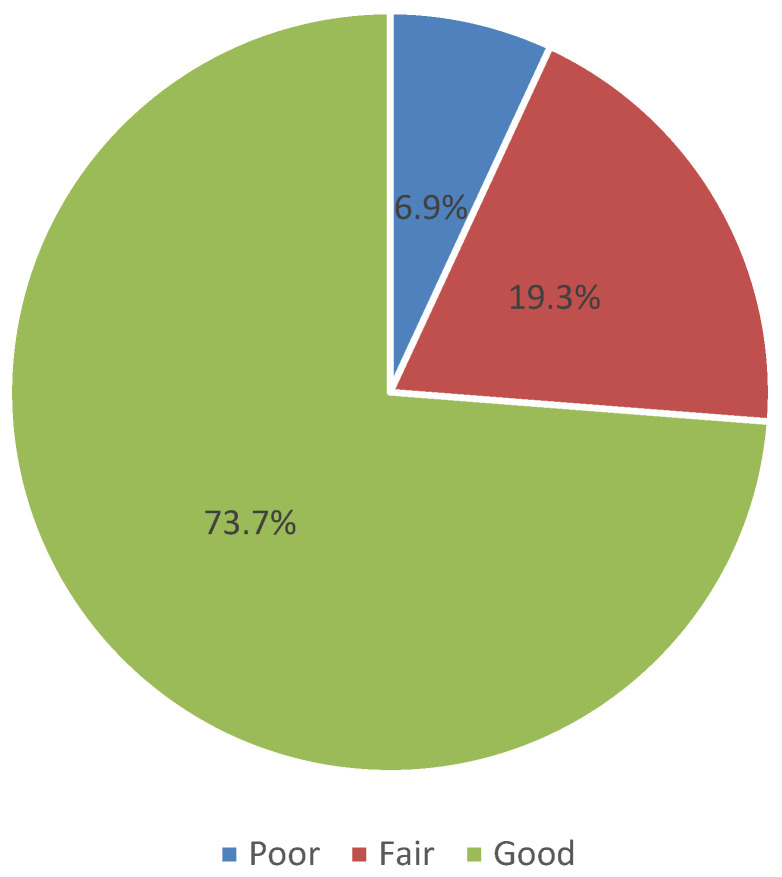
Factors that influence the occurrence of medication administration errors, as perceived by nurses.

Overall, the total reporting practice score averaged 21.0 ± 4.29, reflecting a generally high level of reporting practice. Furthermore, classification of reporting levels showed that the majority of nurses (73.7%) demonstrated “good” reporting practice, while only 6.9% were categorized as having “poor” practice (see also Figure 1).

### 3.3. Assessment of the Factors Contributing to Medication Administration Errors, Under-Reporting of Medication Administration, Nursing Administration Concerns, Reporting Process, and Preventive Measures of Medication Administration Errors

Table 3 illustrates the factors influencing medication administration errors (MAEs). Workload (mean = 4.32 ± 0.90) and insufficient staffing levels (mean = 4.07 ± 1.06) were identified as the most significant contributors, with more than half of the nurses (54.4%) strongly agreeing that workload is a major concern. Other frequently reported factors included illegible handwriting (mean = 3.94) and incorrect medication dispensing by pharmacists (mean = 3.74). In contrast, “nurse incompetence” received the lowest level of agreement (mean = 2.87).

With regard to underreporting, mean scores ranged from 2.65 to 3.21, reflecting a moderate level of concern. Fear of legal consequences was identified as the primary barrier (mean = 3.21 ± 1.27), followed by fear of other negative repercussions (mean = 3.01 ± 1.29).

Regarding administrative responses, nurses moderately agreed that actions taken may not align with the severity of errors (mean = 3.18 ± 1.12) and that there may be a tendency to assign individual blame (mean = 3.38 ± 1.22). The highest level of agreement was observed for the perception that administration considers MAEs as indicators of nursing care quality (mean = 3.64 ± 1.14).

Perceptions of the reporting process were comparatively lower, with mean scores ranging from 2.42 to 3.02. Notably, 41.3% of nurses disagreed with the statement that they would not know how to report errors.

Preventive measures received the highest level of agreement across all domains. A substantial proportion of nurses strongly agreed that adherence to the “five rights” of medication administration (81.1%) and double-checking medications (84.6%) are effective strategies for preventing errors, with mean scores of 4.79 and 4.84, respectively. Continuing education and the presence of pharmacists were also strongly endorsed.

### 3.4. The Correlation Between Medication Administration Error Reporting Practice

Table 4 presents the correlations between medication error reporting practice and the five examined domains. The findings indicate that most correlations were weak and not statistically significant, including factors contributing to MAEs (Rs = 0.033, *p* = 0.601), underreporting factors (Rs = 0.029, *p* = 0.647), nursing administration concerns (Rs = 0.096, *p* = 0.124), and the reporting process (Rs = 0.052, *p* = 0.401).

The only statistically significant relationship was observed between reporting practice and preventive measures, demonstrating a weak but positive correlation (Rs = 0.137, *p* = 0.027). Although the correlation strength is small, its statistical significance indicates a meaningful association between safety-oriented attitudes and reporting behavior.

### 3.5. Association Between Medication Administration Error Reporting Practice and the Socio-Demographic Characteristics

Table 5 examines the association between socio-demographic characteristics and medication error reporting practice. The results indicate that most variables—including age, gender, educational level, marital status, work experience, patient-to-nurse ratio, and training—were not significantly associated with reporting practice (all *p* > 0.05).

However, two variables demonstrated statistically significant relationships. Monthly personal income was significantly associated with reporting practice (Z = 2.257, *p* = 0.024), although median scores were comparable across income groups, suggesting only slight differences in distribution. Similarly, the number of shifts per month showed a significant association (Z = 2.261, *p* = 0.024), with nurses working more than 15 shifts exhibiting marginally higher median reporting scores (21.5 vs. 21).

## 4. Discussion

The present study provides a comprehensive assessment of medication error reporting practices among nurses, including associated factors, perceived barriers, and correlates. Overall, the findings indicate a generally high level of reporting practice, with the majority of nurses (73.7%) demonstrating good reporting behavior and only a small proportion (6.9%) categorized as having poor practice. This suggests the presence of a relatively positive safety culture and reflects an increased awareness of the importance of reporting medication errors as a critical component of patient safety and quality improvement.

The high reporting rates observed—particularly for errors—indicate that many nurses recognize reporting as a professional responsibility. However, variability in reporting behaviors emerged in more complex situations, such as repeated errors or incidents not directly involving the nurse. This inconsistency suggests that while general awareness is high, situational and contextual factors may still influence reporting decisions. This result is consistent with that of [6], which demonstrated a high level of reporting behavior among nurses, aligning with our study’s emphasis on the importance of reporting MAEs promptly. This could be because nurses are highly aware of the significance of reporting MAEs in healthcare settings.

This study found that despite the generally positive reporting practices, several barriers to reporting were identified. Fear of legal consequences and other punitive outcomes emerged as the most prominent deterrents, indicating that concerns about blame and accountability continue to influence reporting behavior. Additionally, nurses perceived that administrative responses may not always align with the severity of errors and may involve assigning individual blame. These findings suggest that a fully established non-punitive or “just culture” may still be lacking, which could limit transparency and hinder effective error reporting.

Preventive measures received the strongest support across all domains, with nurses overwhelmingly endorsing strategies such as adherence to the “five rights” of medication administration, double-checking medications, continuing education, and pharmacist involvement. This finding aligns with our previous observations, indicating that procedures for jointly double-checking medications were widely used and highly regarded as effective in preventing medication administration errors [25]. This strong preventive orientation reflects a proactive approach to patient safety and indicates that nurses are highly engaged in strategies aimed at minimizing errors. Moreover, the only significant correlation identified between reporting practice and preventive measures further supports this finding, suggesting that nurses who prioritize safety practices are more likely to engage in reporting behaviors. Although the correlation was weak, its significance highlights an important link between safety attitudes and reporting culture.

Additionally, the results revealed that most characteristics were not significantly associated with reporting practices, indicating that reporting behavior is relatively consistent across different nurse groups. However, monthly income and number of shifts per month were found to be statistically significant. Nurses with higher workloads (more shifts) demonstrated slightly better reporting practices, which may reflect increased clinical exposure and experience with error recognition. Similarly, the association with income may indirectly relate to experience level, job stability, or organizational position.

This study benefited from a high response rate and diverse representation of nurses across different departments. However, several limitations should be acknowledged, including the reliance on self-reported data, the potential for social desirability bias, and the single-center study design, which may limit the generalizability of the findings.

Future research should include longitudinal studies, recruit participants from multiple healthcare institutions, and examine the impact of educational interventions and policy changes on actual medication error reporting behaviors.

## 5. Conclusions

The purpose of this study was to assess the proportion of MAE reporting by nurses and the associated factors. Compared with previous studies, the findings of this study revealed that medication administration errors were reported at a higher level. The primary factors contributing to these errors were workload and the fear of legal repercussions. At a teaching hospital, 73.7% of nurses consistently reported medication administration errors to the department upon their occurrence. Given that errors are inevitable, this study aims to help healthcare providers comprehend the underlying causes of MAEs and the reasons for their underreporting. Healthcare professionals should consider the potential causes of MAEs and the personal fears of nurses when reporting them. Nurses and all healthcare workers should participate in educational programs to ensure the adoption of best practices. This will ultimately lead to a safer healthcare environment for both patients and staff. Additionally, creating a culture of open communication and support can help reduce the stigma associated with reporting MAEs. By fostering an environment where mistakes are seen as opportunities for learning and improvement, healthcare providers can work together to prevent future errors. Encouraging a blame-free culture will empower nurses to speak up about MAEs without fear of retribution, ultimately improving patient safety outcomes.

## Figures and Tables

**Table 1:** Socio-demographic characteristics.

Study Variables	*N* (%)
Age group	
• 20–25 years	32 (12.4%)
• 26–30 years	50 (19.3%)
• 31–35 years	82 (31.7%)
• 36–40 years	47 (18.1%)
• >40 years	48 (18.5%)
Gender	
• Male	32 (12.4%)
• Female	227 (87.6%)
Educational level	
• Diploma	98 (37.8%)
• Bachelor’s degree	149 (57.5%)
• Master’s degree or PhD	12 (4.6%)
Monthly personal income (SAR)	
• ≤10,000	218 (84.2%)
• 11,000–15,000	30 (11.6%)
• 16,000–20,000	6 (11.6%)
• >20,000	5 (1.9%)
Marital status	
• Single	81 (31.3%)
• Married	172 (66.4%)
• Divorced or widowed	6 (2.3%)
Work experience	
• 1–5 years	73 (28.2%)
• 6–10 years	74 (28.6%)
• 11–15 years	60 (23.2%)
• >15 years	52 (20.0%)
Department patient/nurse ratio	
• 1:1	33 (12.7%)
• 1:2	65 (25.1%)
• 1:3	15 (5.8%)
• 1:4	39 (15.1%)
• 1:5	48 (18.5%)
• 1:6	59 (22.8%)
Number of shifts per month	
• 1–5 shifts/month	24 (9.3%)
• 6–10 shifts/month	6 (2.3%)
• 11–15 shifts/month	43 (16.6%)
• More than 15 shifts/month	186 (71.8%)
Have you received any training on administrative techniques and errors?	
• Yes	230 (88.8%)
• No	29 (11.2%)

**Table 2:** Assessment of medication administration error reporting practice (*n* = 259).

Statement	Never *N* (%)	Rarely *N* (%)	Sometimes *N* (%)	Often *N* (%)	Always *N* (%)	Mean ± SD
When a medication administration error occurs, I report it to the department	9 (3.5%)	5 (1.9%)	11 (4.2%)	3 (1.2%)	231 (89.2%)	4.71 ± 0.91
2. I report medication administration errors even if they do not harm the patient	8 (3.1%)	5 (1.9%)	16 (6.2%)	7 (2.7%)	223 (86.1%)	4.67 ± 0.91
3. I report medication administration errors even if it is not possible to improve the patient’s health status subsequent to that error	9 (3.5%)	4 (1.5%)	17 (6.6%)	6 (2.3%)	223 (86.1%)	4.66 ± 0.93
4. I report medication administration errors only when similar errors have occurred previously in the department	103 (39.8%)	12 (4.6%)	21 (8.1%)	16 (6.2%)	107 (41.3%)	3.05 ± 1.83
5. I report medication administration errors even if I was not involved in them	39 (15.1%)	12 (4.6%)	33 (12.7%)	17 (6.6%)	158 (61.0%)	3.94 ± 1.51
**Total medication error reporting practice score**	--	--	--	--	--	**21.0 ± 4.29**
Level of ME reporting practice						***N* (%)**
• Poor (score ≤ 50%)	--	--	--	--	--	18 (6.9%)
• Fair (score 51–74%)	--	--	--	--	--	50 (19.3%)
• Good (score ≥ 75%)	--	--	--	--	--	191 (73.7%)

Response has ranged from “Never” coded with 1 to “Always” coded with 5.

**Table 3:** Assessment of the factors contributing to medication administration errors, under-reporting of medication administration, nursing administration concerns, reporting process, and preventive measures of medication administration errors (*n* = 259).

Factors Contributing to Medication Administration	SD *N* (%)	D *N* (%)	N *N* (%)	A *N* (%)	SA *N* (%)	Mean ± SD
Lack of sufficient training for nurses	44 (17.0%)	63 (24.3%)	44 (17.0%)	49 (18.9%)	59 (22.8%)	3.06 ± 1.42
2. Workload (high patient/nurse ratio)	3 (1.2%)	10 (3.9%)	28 (10.8%)	77 (29.7%)	141 (54.4%)	4.32 ± 0.90
3. Unreadable handwriting by prescribers	7 (2.7%)	21 (8.1%)	42 (16.2%)	99 (38.2%)	90 (34.7%)	3.94 ± 1.04
4. Wrong prescription order by the physician	12 (4.6%)	34 (13.1%)	55 (21.2%)	85 (32.8%)	73 (28.2%)	3.67 ± 1.15
5. Insufficient staffing	8 (3.1%)	16 (6.2%)	41 (15.8%)	79 (30.5%)	115 (44.4%)	4.07 ± 1.06
6. Nurse incompetence (lack of appropriate knowledge and skills)	51 (19.7%)	63 (24.3%)	57 (22.0%)	45 (17.4%)	43 (16.6%)	2.87 ± 1.36
7. Interruption during medication administration	11 (4.2%)	33 (12.7%)	54 (20.8%)	85 (32.8%)	76 (29.3%)	3.70 ± 1.15
8. Wrong dispensing by pharmacists	9 (3.5%)	26 (10.0%)	60 (23.2%)	93 (35.9%)	71 (27.4%)	3.74 ± 1.08
**Total score**	**--**	**--**	**--**	**--**	**--**	**29.4 ± 6.31**
**Factors associated with under-reporting of medication administration errors**						
I would be viewed as incompetent by colleagues	42 (16.2%)	68 (26.3%)	58 (22.4%)	66 (25.5%)	25 (9.7%)	2.86 ± 1.24
2. I would be discriminated against by coworkers	48 (18.5%)	86 (33.2%)	53 (20.5%)	53 (20.5%)	19 (7.3%)	2.65 ± 1.21
3. Other employees in the hospital would become aware of my administrative errors	46 (17.8%)	69 (26.6%)	54 (20.8%)	59 (22.8%)	31 (12.0%)	2.85 ± 1.29
4. It is likely that I would face repercussions	41 (15.8%)	52 (20.1%)	68 (26.3%)	60 (23.2%)	38 (14.7%)	3.01 ± 1.29
5. It is possible I may face a lawsuit or legal action	35 (13.5%)	39 (15.1%)	66 (25.5%)	75 (29.0%)	44 (17.0%)	3.21 ± 1.27
**Total score**	**--**	**--**	**--**	**--**	**--**	**14.6 ± 5.19**
**Nursing administration concerns**						
I would receive negative feedback from nursing administration if I were to report a medication administration error	36 (13.9%)	73 (28.2%)	47 (18.1%)	69 (26.6%)	34 (13.1%)	2.97 ± 1.28
2. Nursing administration believes that medication administration errors are a measure of the quality of nursing care provided	15 (5.8%)	31 (12.0%)	48 (18.5%)	104 (40.2%)	61 (23.6%)	3.64 ± 1.14
3. The response toward staff by nursing administration would not match the severity of the medication administration errors	20 (7.7%)	53 (20.5%)	76 (29.3%)	80 (30.9%)	30 (11.6%)	3.18 ± 1.12
4. Nursing administration would focus on the individual nurse as the primary cause of the medication error	18 (6.9%)	53 (20.5%)	55 (21.2%)	79 (30.5%)	54 (20.8%)	3.38 ± 1.22
**Total score**	**--**	**--**	**--**	**--**	**--**	**13.2** **± 3.66**
**Reporting process**						
Incident report forms are too complicated	18 (6.9%)	86 (33.2%)	63 (24.3%)	58 (22.4%)	34 (13.1%)	3.02 ± 1.17
2. Incident reporting wastes too much time	24 (9.3%)	95 (36.7%)	56 (21.6%)	65 (25.1%)	19 (7.3%)	2.85 ± 1.12
3. I would not know how to report medication administration errors if they occurred	59 (22.8%)	107 (41.3%)	32 (12.4%)	46 (17.8%)	15 (5.8%)	2.42 ± 1.19
**Total score**	**--**	**--**	**--**	**--**	**--**	**8.29 ± 2.82**
**Preventive measures of medication administration errors**						
Following the five rights (right patient, right drug, right route, right dose, right time) prevents drug administration errors from occurring	0	1 (0.4%)	3 (1.2%)	45 (17.4%)	210 (81.1%)	4.79 ± 0.46
2. Double-checking of medications should be practiced to prevent medication administration errors	0	0	1 (0.4%)	39 (15.1%)	219 (84.6%)	4.84 ± 0.38
3. It is important for nurses to have a pharmacist working on the ward to prevent medication administration errors	9 (3.5%)	26 (10.0%)	48 (18.5%)	54 (20.8%)	122 (47.1%)	3.98 ± 1.17
4. It is important for nurses to have continuing education to prevent medication administration errors	1 (0.4%)	4 (1.5%)	13 (5.0%)	74 (28.6%)	167 (64.5%)	4.55 ± 0.69
5. A computerized system should be used for administering drugs to prevent medication administration errors	10 (3.9%)	17 (6.6%)	60 (23.2%)	66 (25.5%)	106 (40.9%)	3.93 ± 1.12
**Total score**	**--**	**--**	**--**	**--**	**--**	**22.1 ± 2.58**

SD—Strongly disagree; D—Disagree; N—Neutral; A—Agree; SA—Strongly agree. Response ranged from “strongly disagree” coded with 1 to “strongly agree” coded with 5.

**Table 4:** Spearman correlation coefficient between medication error reporting practice and factors contributing to medication administration, under-reporting of medication administration, nursing administration concerns, reporting process, and preventive measures of medication administration errors (*n* = 259).

Domain	ME Reporting Practice	
	Rs-Value	*p*-Value
Factors contributing to the medication administration score	0.033	0.601
Factors associated with under-reporting of medication administration score	0.029	0.647
Nursing administration concerns score	0.096	0.124
Reporting process score	0.052	0.401
Preventive measures of medication administration errors score	0.137	0.027 **

** Significant at *p* < 0.05 level.

**Table 5:** Association between medication administration error reporting practice and the socio-demographic characteristics of the nurses (*n* = 259).

Factor	ME Reporting Practice Score (25) Median (IQR)	Z-Statistic	*p*-Value ^§^
Age group			
• ≤35 years	21 (5.0)	1.315	0.189
• >35 years	21 (7.0)		
Gender			
• Male	21 (8.0)	1.074	0.283
• Female	21 (6.0)		
Educational level			
• Diploma	22 (5.25)	0.505	0.614
• Bachelor’s degree or higher	21 (6.0)		
Monthly personal income (SAR)			
• ≤10,000	21 (5.25)	2.257	0.024 **
• >10,000	21 (3.50)		
Marital status			
• Unmarried	21 (6.0)	0.202	0.840
• Married	21 (5.0)		
Work experience			
• ≤10 years	21 (5.0)	0.570	0.569
• >10 years	21 (7.0)		
Department patient/nurse ratio			
• 1:1–1:3	21 (7.0)	0.853	0.394
• 1:4–1:6	21 (5.0)		
Number of shifts per month			
• ≤15 shifts/month	21 (4.50)	2.261	0.024 **
• >15 shifts/month	21.5 (5.0)		
Have you received any training on administrative techniques and errors?			
• Yes	21 (6.0)	0.712	0.476
• No	17.3 (8.0)		

^§^ *p*-value has been calculated using Mann–Whitney Z-test. ** Significant at *p* < 0.05 level.

## Data Availability

The original contributions presented in this study are included in the article. Further inquiries can be directed to the corresponding author(s).
